# Early Prediction of Delirium in Postcardiac Surgery Patients: Machine Learning Model Development and External Validation

**DOI:** 10.2196/73283

**Published:** 2026-02-11

**Authors:** Huixiu Hu, Yuxiang Wang, Houfeng Li, Qinglai Zang, Jing Huang, Ying Zhang, Jinjing Wu, Long Liu, Zhen Xing, Yaohua Yu

**Affiliations:** 1 The Graduate School of Fujian Medical University Fuzhou, Fujian China; 2 Department of Anesthesiology, The First Hospital of Putian City Putian China; 3 Department of Anesthesiology The First Affiliated Hospital of Hebei North University Zhangjiakou, Hebei China; 4 School of Health Science and Engineering University of Shanghai for Science and Technology Shanghai, Shanghai China; 5 Department of Anesthesiology Changhai Hospital, the Second Military Medical University Shanghai China; 6 School of Anesthesiology Second Military Medical University Shanghai China; 7 Information Center The Second Affiliated Hospital of Naval Medical University Shanghai China; 8 Graduate School, Wannan Medical College Wuhu, Anhui China

**Keywords:** prediction model, machine learning, cardiac surgery, intensive care unit, delirium, Medical Information Mart for Intensive Care IV version 2.0 database, MIMIC-IV 2.0 database, eICU Collaborative Research Database, eICU-CRD

## Abstract

**Background:**

Delirium is a frequent postoperative complication among patients who have undergone cardiac surgery and is associated with prolonged hospitalization, cognitive decline, and increased mortality. Early prediction of delirium is therefore critical for initiating timely interventions.

**Objective:**

This study proposes the development and validation of a machine learning–based model to predict postoperative delirium in patients undergoing cardiac surgery during intensive care unit (ICU) care, facilitating the early detection of individuals at high risk of delirium and supporting clinicians in the deployment of targeted preventive strategies.

**Methods:**

This study extracted data on postoperative cardiac surgery patients who remained in the ICU for more than 24 hours from the Medical Information Mart for Intensive Care IV version 2.0 (MIMIC-IV 2.0) database and the eICU Collaborative Research Database (eICU-CRD). The MIMIC-IV 2.0 cohort was randomly divided into a training set and an internal validation set in a 7:3 ratio, whereas the eICU-CRD functioned as an independent validation cohort. We used data from the first 24 hours of ICU monitoring to model the likelihood of delirium over the entire ICU admission period. Delirium was identified by a positive Confusion Assessment Method for the Intensive Care Unit evaluation (ie, score ≥4). We built predictive models by using logistic regression, support vector classifier, extreme gradient boosting (XGB), and random forest classifiers. Their performance was assessed via the area under the receiver operating characteristic curve, accuracy, sensitivity, positive predictive value, negative predictive value, and *F*_1_-score.

**Results:**

The analysis involved 2124 patients from the MIMIC-IV 2.0 database and 2406 from the eICU-CRD. A set of 57variables was selected to construct the predictive models. Among the various machine learning models tested, the XGB model demonstrated the best performance for delirium prediction during internal validation. As for external validation, the model achieved an area under the receiver operating characteristic curve of 0.75, indicating strong discriminatory ability. The most important predictive features identified by the model included hospital length of stay, minimum Glasgow Coma Scale score, mean blood pressure, Sequential Organ Failure Assessment score, weight, urine output, heart rate, and age.

**Conclusions:**

The XGB model with strong predictive capability for ICU delirium after cardiac surgery was developed and externally validated. This model offers essential technical support for building real-time delirium alert systems and enables ongoing risk stratification and evidence-based decision-making within the ICU environment.

## Introduction

Delirium is an acute neuropsychiatric syndrome commonly associated with encephalopathy, acute cerebral dysfunction, and states of confusion, particularly following surgical procedures [1-4]. In patients undergoing cardiac surgery, the incidence of postoperative delirium has been reported to range from 10% to 40% [5-7]. This condition is linked to a variety of adverse outcomes, including heightened pain perception, depression, cognitive impairment, and increased mortality [8,9]. Currently, the assessment of patients’ arousal can be conducted using standardized tools such as the Richmond Agitation-Sedation Scale and the Confusion Assessment Method for the Intensive Care Unit (CAM-ICU) to identify different types of delirium with distinct characteristics [10-12]. Despite the existence of standardized instruments, the diagnosis of delirium frequently depends on the patient’s subjective assessment of their condition. If the occurrence of delirium in patients could be predicted within a short period, it will substantially reduce the aforementioned risks.

Machine learning (ML), a branch of artificial intelligence, has driven notable progress across numerous domains of health care [13,14]. One such area where ML has shown its potential is in the postoperative surveillance with cardiac surgery, offering more information to predict delirium [15,16]. It has the potential to improve patient health care outcomes [17,18]. Compared to traditional data analysis techniques, ML models can provide more intricate predictions and perform real-time monitoring using objective data from all patients [19-21]. Furthermore, recent research has also used ML to forecast the near-term mortality rates of patients after cardiac operations. For example, Nistal-Nuño [22] constructed an extreme gradient boosting (XGB)–based predictive model to estimate 24-hour postoperative mortality following cardiac surgery. The outcome demonstrated that XGB attained an area under the receiver operating characteristic curve (AUC) of 87.5%, signifying the model’s exceptional performance in forecasting intensive care unit (ICU) mortality, notably surpassing other models. Zhang et al [23] assessed an ML model, comparing it to existing severity-of-illness systems to develop a real-time tool for predicting death. However, no correlation-predictive models using ML have been developed for patients who experience delirium after cardiac surgery.

In this study, we created and verified 4 models using ML techniques to anticipate the occurrence of delirium and facilitate identification. Furthermore, we enhanced the interpretability of the results by prioritizing the independent variables according to their predictive significance.

## Methods

### Ethical Considerations

This study used data from 2 publicly available critical care databases: Medical Information Mart for Intensive Care IV version 2.0 (MIMIC-IV 2.0) database and eICU Collaborative Research Database (eICU-CRD). Both databases were approved by the institutional review boards of the Beth Israel Deaconess Medical Center and the Massachusetts Institute of Technology. As all data were fully deidentified before release, the requirement for individual informed consent was waived in accordance with the Declaration of Helsinki and applicable regulations. All team members underwent certified training in “Data or Specimens Only Research” to comply with ethical regulations governing dataset access.

### Study Population

This study used 2 publicly available critical care databases to develop and validate a predictive model. The training dataset was sourced from MIMIC-IV 2.0, which includes 76,943 ICU admissions recorded at Beth Israel Deaconess Medical Center (Boston, Massachusetts, United States) between 2008 and 2019 [24]. For external validation, we used the eICU-CRD, which contains deidentified data for over 200,000 patients admitted to 208 US hospitals between 2014 and 2015 [25]. Both databases contain structured clinical data, including demographics, vital signs, laboratory test results, procedures, medications, and outcomes. We included adult patients (aged ≥18 years) who underwent major cardiovascular surgeries, such as coronary artery bypass grafting, heart valve repair or replacement, combined procedures, or other surgeries involving cardiopulmonary bypass.

**Figure 1:**
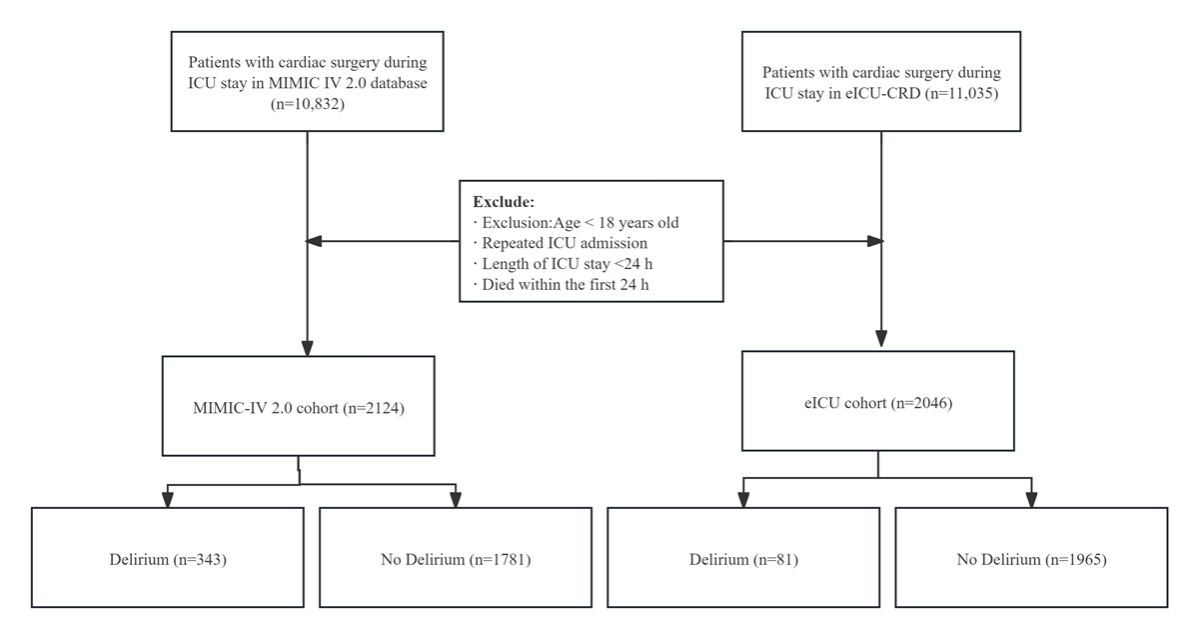
Schematic representation of the study design. eICU-CRD: eICU Collaborative Research Database; ICU: intensive care unit; MIMIC-IV 2.0: Medical Information Mart for Intensive Care IV version 2.0.

We applied consistent inclusion and exclusion criteria across both datasets to ensure cohort comparability and data quality. Inclusion criteria required patients to meet the age threshold and have documented cardiovascular surgery. We excluded patients who had ICU stays shorter than 24 hours (to ensure sufficient observation data), missing essential demographic or outcome variables, or delirium recorded within the first 24 hours of ICU admission (to preserve the prediction time window). Although the 2 datasets differ in time range and hospital coverage, we aligned the study population by applying uniform definitions for surgical type and using a standardized 24-hour observation window. Figure 1 summarizes the baseline characteristics of the 2 cohorts.

### Delirium Assessment

Delirium served as the primary outcome, identified on the basis of a positive CAM-ICU assessment (score ≥4) and consistent diagnostic coding. The observation window was the first 24 hours after ICU admission, during which patient data were collected for modeling. If at least one positive delirium assessment occurred at the time of prediction, the patient was considered delirious.

### Data Extraction and Processing

The clinical data were retrieved and extracted using the structured query language, with pgAdmin 4 serving as the administrative platform for PostgreSQL. The prediction model included just clinical and laboratory characteristics that were accessible on the initial day of admission to the ICU, with patients being recognized by their unique ID numbers. The predictors consisted of the following variables— (1) demographics: age, gender, ethnicity and weight; (2) vital signs: heart rate, mean blood pressure, respiratory rate, systolic blood pressure, and temperature; (3) laboratory analysis: hemoglobin level, platelet count, white blood cell count, lactate and urine output; (4) severity scoring: Glasgow Coma Scale (GCS) and Sequential Organ Failure Assessment (SOFA) scores; (5) comorbidities: myocardial infarction, congestive heart failure, peripheral vascular disease, cerebrovascular disease, dementia, chronic pulmonary disease, rheumatic disease, peptic ulcer disease,diabetes (with control, without control), paraplegia, renal disease, malignant cancer, severe liver disease, and AIDS; (6) medications: opioids, barbiturates, benzodiazepines, acetaminophen, antipsychotics, anticoagulant, antihistamines, diuretics, anesthesia, and anticholinergics; (7) treatment measures: emergency admission, first care unit, last care unit, renal replacement therapy, invasive ventilation, length of ICU stay.

### Missing Data Management

Variables exhibiting a missing value rate exceeding 10% were omitted to prevent potential bias. Variables with less than 10% missing values were subjected to multivariable imputations [26].

### Data Balance

The dataset showed a marked imbalance, with notably fewer positive delirium cases compared with negative ones, which caused the model to lean toward predicting the majority (negative) class. To mitigate this issue, we used the Synthetic Minority Oversampling Technique to artificially augment the number of positive samples, thereby achieving a more balanced class distribution and enhancing the model’s ability to generalize.

### Feature Selection

The feature selection process involved using the recursive elimination of features approach of the random forest [27]. This method was used to identify the most optimal combinations of predictive variables. By examining the weight of features and their correlation and after stratified 10-fold cross-validation, final features were selected based on importance scores, correlation analysis, and cross-validation results. This process reduced dimensionality while preserving predictive power.

### Model Development and Hyperparameter Tuning

The MIMIC-IV 2.0 dataset (N=2124) was randomly split into a training set (1487/2124, 70%) and a testing set (637/2124, 30%), whereas the eICU-CRD dataset was used as an external validation cohort. We developed prediction models using 4 widely adopted ML algorithms. Logistic regression was implemented for binary classification using maximum likelihood estimation [28]. Random forest, an ensemble learning method, combined multiple decision trees through majority voting to enhance predictive performance [29]. XGB used a gradient boosting framework to iteratively build strong learners from weak ones [30]. Support vector classifier aimed to find the optimal hyperplane in a high-dimensional space for classification [31]. Bayesian optimization was used to identify optimal hyperparameters for each model, improving training efficiency and performance [32].

### Model Performance Evaluation

To comprehensively assess the discriminatory performance of the prediction models, we used the receiver operating characteristic (ROC) as the primary evaluation metric. Additional metrics included accuracy, positive predictive value, negative predictive value, and sensitivity. We also reported the *F*_1_-score, the harmonic mean of precision and sensitivity, to reflect the balance between these two metrics. Together, these indicators were used to evaluate the clinical applicability of each model in stratifying the risk of postoperative delirium among cardiac surgery patients. Shapley Additive Explanations (SHAP) was used to investigate the interpretability of the final predictive model.

### Statistical Analysis

All statistical analyses were conducted using Stata 17.0 and SPSS (version 27.0; IBM Corp). Frequencies and percentages were used to summarize categorical variables, with comparisons made via the chi-square test. The distribution of continuous variables was assessed using the Shapiro-Wilk test. Normally distributed data were reported as mean (SD) and compared using independent 2-tailed *t* tests. Skewed data were summarized as median and IQR and analyzed using the Mann-Whitney U or Kruskal-Wallis test, based on group composition. Statistical significance was determined using 2-sided tests, with a threshold of *P*<.05.

## Results

### Baseline Characteristics

A total of 2124 patients from the MIMIC-IV 2.0 database were included in the final analysis. Among them, 16.1% (343/2124) of cardiac surgery patients were diagnosed with delirium during their hospital stay, occurring after the first day of ICU admission. In the external validation cohort, an analysis was conducted on 2046 cases obtained from the eICU-CRD, of whom 3.81% (81/2046) developed delirium during the same postoperative period, also defined as after the first ICU day. In these patients with delirium, maximum heart rate, minimum mean blood pressure, minimum hemoglobin level, minimum platelet count, maximum white blood cell count, urine output, minimum GCS score, SOFA, and length of ICU stay showed a notable disparity between the two different groups. Tables 1-3 present a concise summary of the comparison of fundamental traits, vital signs, and laboratory analysis between patients with and without delirium. According to the data, patients with delirium are predominantly male, typically older, and have longer hospital stays with higher severity scores upon admission. Additionally, factors such as weight loss, decreased urine output, and decreased mean arterial pressure may all exacerbate the likelihood of delirium in patients.

**Table 1:** Characteristics of patients and controls from the development dataset for the first 24-hour model cohort: demographics and vital signs.

Patient characteristics			MIMIC-IV 2.0^a^ cohort									eICU-CRD^b^ cohort						
			No delirium (n=1781)		Delirium (n=343)		*P* value			No delirium (n=1965)				Delirium (n=81)			*P* value	
**Gender, n (%)**								.10										.09
	Male	1144.0 (64.2)		195.0 (56.9)					937.0 (49.5)				46 (57)					
	Female	637.0 (35.8)		148.0 (43.1)					992.0 50.5)				35 (43)					
**Race, n (%)**								.80										.62
	Asian	47.0 (2.6)		6.0 (1.7)					29.0 (1.5)				6.0 (1.4)					
	Black	80.0 (4.5)		19.0 (5.5)					475.0 (24.2)				18.0 (22.5)					
	Hispanic	126.0 (7.1)		42.0 (12.2)					1227.0 (62.4)				53.0 (65)					
	White	1391.0 (78.1)		219.0 (63.8)					132.0 (6.7)				12.0 (14.8)					
	Unknown	29.0 (1.6)		47.0 (13.7)					96.0 (4.9)				10.0 (2.3)					
	Other	108.0 (6.1)		10.0 (2.)					6.0 (0.3)				2.0 (2.5)					
Age (y), median (IQR)			70.0 (61.0-79.0)		75.0 (64.0-81.0)		<.001			68.0 (57.0-77.0)				71.0 (61.0-79.8)			<.001	
Weight (kg), median (IQR)			83.8 (70.8-96.7)		80.0 (60.0-95.4)		.02			85.2 (70.0-102.0)				84.5 (67.9-103.4)			.31	
**Vital signs, median (IQR)**																		
	Heart rate min (bpm)	67.0 (60.0-74.0)		68.0 （59.0-77.0）		.12			69.0（59.0-80.0）				70.0（60.0-80.0）			.29		
	Heart rate max (bpm)	94.0 (86.0-106.0)		97.0 （88.0-110.0）		<.001			101.0（88.0-116.0）				107.0 (92.2-124.5)			<.001		
	Heart rate mean (bpm)	80.2 (73.3-87.5)		82.7 (75.6-89.3)		<.001			83.6 (75.7-94.4)				86.3 (75.7-97.9)			.007		
	Mean blood pressure min (mm Hg)	57.0 (53.0-62.0)		55.0 (49.5-60.0)		<.001			62.0 (54.0-72.0)				60.0 (52.0-68.0)			<.001		
	Mean blood pressure max (mm Hg)	97.0 (89.0-107.0)		95.0 (88.0-108.0)		.33			103.0 (99.0-118.0）				104.0 (91.0-122.0)			.31		
	Mean blood pressure mean (mm Hg)	74.6 (70.3-79.4)		73.3 (69.3-78.8)		.02			80.0 (76.6-90.4)				78.2 (70.3-88.2)			.02		
	Respiratory rate min (bpm)	12.0 (10.0-14.0)		12.0 (9.0-14.0)		.75			13.0 (11.0-16.0)				13.0 (11.0-16.0)			.63		
	Respiratory rate max (bpm)	26.0 (23.0-29.0)		26.0 (23.00-30.0)		.69			27.0 (24.0-32.00)				28.0 (24.0-33.00)			.42		
	Respiratory rate mean (bpm)	17.8 (16.2-19.6)		18.1 (16.2-20.2)		.23			19.2 (17.1-21.9)				19.2 (16.9-22.9)			.76		
	Systolic blood pressure min (mm Hg)	93.0 (91.0-95.0)		93.0 (91.00-96.0)		.85			97.0 (95.6-98.4)				97.1 (95.34-98.63)			.001		
	Systolic blood pressure max (mm Hg)	91.0 (93.0-95.0)		93.0 (91.0-96.0)		.003			92.0 (89.0-94.0)				91.0 (86.3-94.0)			.15		
	Systolic blood pressure mean (mm Hg)	97.7 (96.5-98.7)		97.9 (96.7-99.0)		.14			100.0 (99.0-100.0)				100.0 (100.0-100.0)			.92		
	Temperature mean (℃)	36.7 (36.5-36.9)		36.72 (36.5-37.0)		.48			36.7 (36.6-37.0)				36.8 (36.9-36.5)			.007		

^a^MIMIC-IV Medical Information Mart for Intensive Care IV version 2.0.

^b^eICU-CRD: eICU Collaborative Research Database.

**Table 2:** Characteristics of patients and controls from the development dataset for the first 24-hour model cohort: laboratory test results and comorbidities.

Patient characteristics			MIMIC-IV 2.0^a^ cohort							eICU-CRD^b^ cohort				
			No delirium (n=1781)		Delirium (n=343)		*P* value		No delirium (n=1965)			Delirium (n=81)		*P* value
**Laboratory results, median (IQR)**														
	Hemoglobin min (g/100 mL)	8.4 (7.5-9.6)		9.0 (7.9-10.3)		<.001		8.4 (7.3-9.4)			9.0 (7.9-10.3)		<.001	
	Hemoglobin max (g/100 mL)	11.5 (10.0-12.9)		11.4 (10.4-14.2)		.64		11.4 (10.4-12.9)			11.4 (10.4-20.1)		.62	
	Platelet min (10^9^/L)	147.0 (111.0-198.0)		130.0 (95.0-179.0)		<.001		145.0 (109.0-197.0)			128.0 (96.0-178.0)		<.001	
	Platelet count max (10^9^/L)	125.0 (151.0-245.0)		188.0 (149.0-242.0)		.57		128.0 (97.0-235.0)			186.0 (153.0-245.0)		.55	
	White blood cell min (10^9^/L)	8.8 (6.3-11.7)		9.5 (6.9-12.3)		.12		9.2 (6.3-12.0)			9.6 (7.0-12.4)		.14	
	White blood cell max (10^9^/L)	13.1 (10.0-17.3)		14.8 (11.3-19.6)		<.001		14.0 (10.0-17.3)			14.7 (11.4-20.0)		<.001	
	Lactate min (mmol/L)	1.2（0.9-1.5）		1.2（0.9-1.6）		.21		1.4 (1.0-2.1)			1.3 (0.9-1.9)		.18	
	Urine output (mL)	1832.0 (1290.0-2617.0)		1575.0 (1002-2321.0)		<.001		1496.0 (796.3-2600.0)			1229.5 (600.0-2056.0)		<.001	
**Comorbidity, n (%)**														
	Myocardial infarction	30 (1.7)		3 (0.9)		.37		181 (9.2)			30 (6.8)		.11	
	Congestive heart failure	724 (40.7)		184 (53.6)		<.001		1251 (63.7)			51 (63)		.78	
	Peripheral vascular disease	281 (15.8)		84 (24.5)		<.001		14 (0.7)			1.0 (1.1)		.36	
	Cerebrovascular disease	186 (10.4)		65 (19.0)		<.001		90 (4.6)			6 (7.3)		.02	
	Chronic pulmonary disease	498 (28)		125 (36.4)		.002		303 (15.4)			11 (13.6)		.35	
	Renal disease	387 (21.7)		96 (28.0)		.01		321 (16.3)			13 (15.7)		.74	
	Diabetes with control	1538 (7.7)		30 (8.7)		.53		155 (7.9)			7 (8.5)		.58	
	Diabetes without control	495 (27.8)		97 (28.3)		.85		503 (30.2)			24 (29.5)		.84	
	Rheumatic disease	78 (4.4)		23 (6.7)		.06		82.0 (4.6)			5 (6.5)		.06	
	Peptic ulcer disease	13 (0.7)		7 (2)		.02		9.0 (0.5)			1 (1.5)		.02	
	Severe liver disease	16 (0.9)		7 (2)		.06		18.0 (1.0)			2 (2.3)		.07	
	Dementia	11 (0.6)		3 (0.9)		.59		13.0 (0.7)			1 (0.9)		.61	
	Paraplegia	15 (0.8)		12 (3.5)		<.001		16.0 (0.9)			3 (3.7)		<.001	
	Malignant cancer	103 (5.8)		16 (4.7)		.41		181.0 (9.2)			5 (6.8)		.11	
	AIDS	4 (0.2)		5 (3)		.38		1964.00 (99.0)			1 (1.2)		.04	

^a^MIMIC-IV Medical Information Mart for Intensive Care IV version 2.0.

^b^eICU-CRD: eICU Collaborative Research Database.

**Table 3:** Characteristics of patients and controls from the development dataset for the first 24-hour model cohort: score, drug, and treatment measures.

Patient characteristics		MIMIC-IV 2.0^a^ cohort				eICU-CRD^b^ cohort		
		No delirium (n=1781)	Delirium (n=343)	*P* value	No delirium (n=1965)		Delirium (n=81)	*P* value
**Score, median (IQR)**								
	GCS^c^ (min)	14.0 (14.0-15.0)	11.0 (6.0-14.0)	<.001	15.0 (14.0-15.00)		12.0 (8.0-14.0)	<.001
	SOFA^d^	4.0 (2.0-7.0)	8.0 (6.0-11.0)	<.001	5.0 (3.0-7.0)		7.0 (5.0-10.0)	<.001
**Drug, n (%)**								
	Acetaminophen	1005 (56.4)	164 (47.8)	.003	1104 (56.2)		41 (50.6)	.054
	Anesthesia	38 (2.1)	5 (1.5)	.002	35 (1.9)		1 (1.2)	.02
	Anticholinergics	1062 (59.6)	221 (64.4)	.10	387 (19.7)		19 (23.5)	.03
	Anticoagulant	519 (29.1)	92 (26.8)	.39	539 (27.4)		36 (44.4)	.41
	Antipsychotics	18 (1.0)	11 (3.2)	.001	21 (1.1)		17 (3.9)	<.001
	Barbiturates	2.0 (0.1)	343 (100)	.54	3 (0.1)		81 (100)	.54
	Benzodiazepines	138 (7.7)	32 (9.3)	.32	142 (8)		35 (9.5)	.34
	Diuretics	694 (39)	131 (38.2)	.79	854 (43.5)		29 (35.5)	.008
	Opioids	1040 (58.4)	201 (58.6)	.94	981 (49.9)		38 (46.9)	.28
**Treatment measures, n (%)**								
	Emergency admission	1121 (62.9)	231 (67.3)	.12	380 (19.3)		17 (20.9)	.52
	First care unit	1724 (96.8)	321 (93.6)	.004	1779 (90.5)		75 (92.6)	.04
	Last care unit	42 (2.4)	16 (4.7)	.02	453 (23)		36 (44.4)	<.001
	Renal Replacement Therapy	15 (0.8)	11 (3.2)	<.001	719 (36.6)		46 (56.8)	.047
	Invasive ventilation	1179 (66.2)	298 (86.9)	<.001	1699 (86.5)		77 (95.1)	<.001
Length of ICU^e^ stay, median (IQR)		2.4 (2.0-3.4)	5.9（4.0-10.3）	<.001	3.20 (3.0-5.5)		6.7 (5.3-12.5)	<.001

^a^MIMIC-IV Medical Information Mart for Intensive Care IV version 2.0.

^b^eICU-CRD: eICU Collaborative Research Database.

^c^GCS: Glasgow Coma Scale.

^d^SOFA: Sequential Organ Failure Assessment.

^e^ICU: intensive care unit.

### Model Performance Evaluation

**Figure 2:**
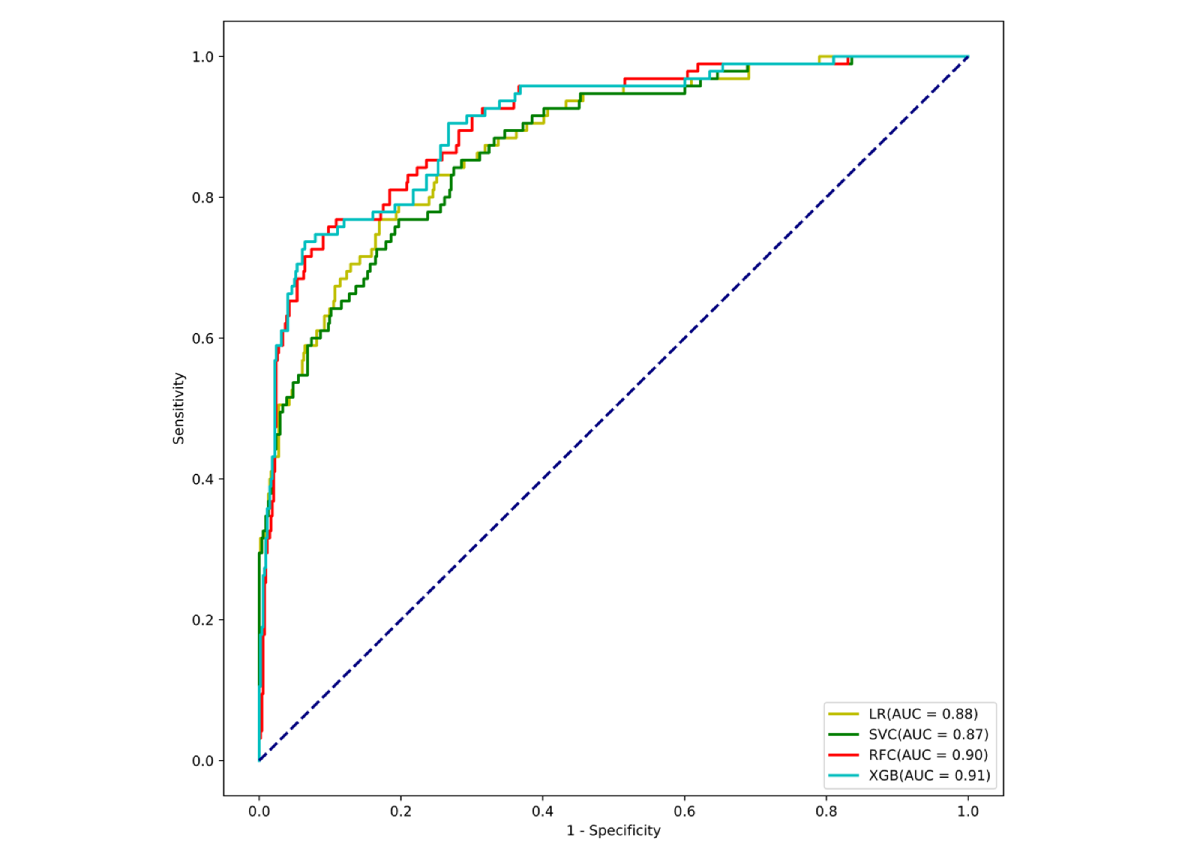
Receiver operating characteristic curves of different machine learning algorithms evaluated on the internal validation set. LR: logistic regression; SVC: support vector classifier; RFC: random forest classifier; XGB: extreme gradient boosting.

**Figure 3:**
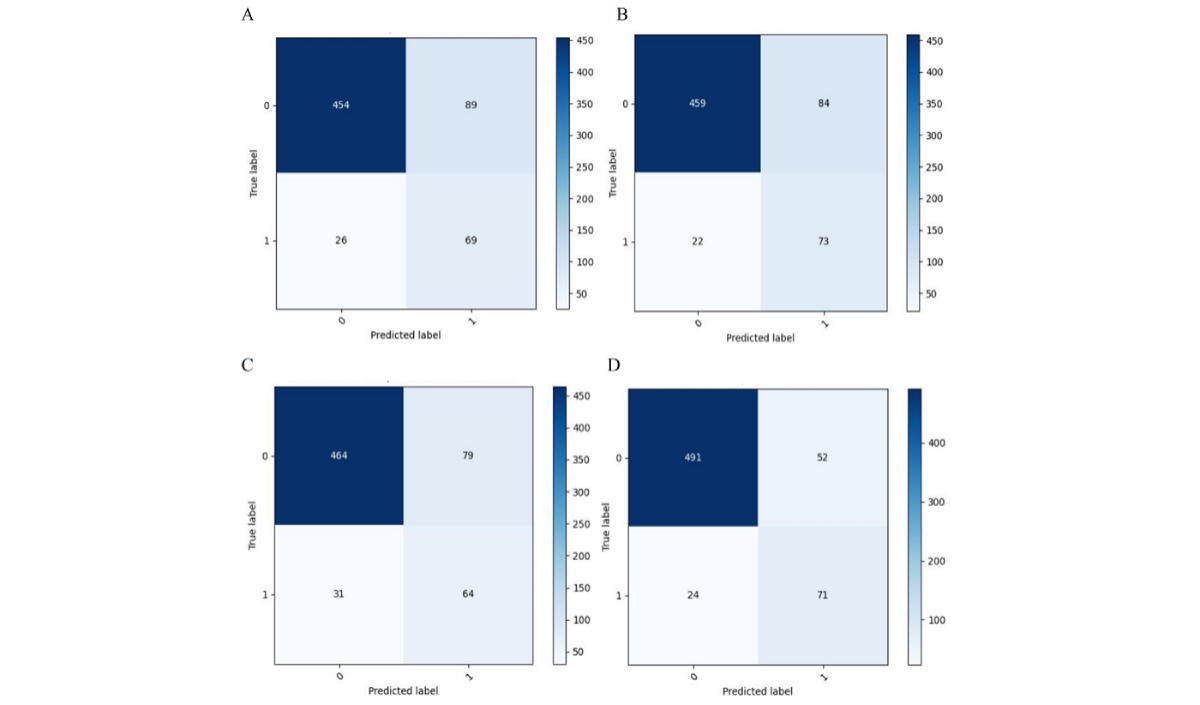
Confusion matrix for binary classification. (A) Logistic regression (LR), (B) extreme gradient boosting (XGB), (C) support vector classifier (SVC), (D) random forest classifier (RFC).

Using 4 ML algorithms, we developed predictive models to assess the risk of postoperative delirium in cardiac surgery patients, leveraging electronic health record data for early identification of high-risk individuals. Figure 2 presents ROC curves of all models, allowing a systematic comparison of their discriminative performance. Among the models, XGB demonstrated the best overall predictive performance, achieving the highest AUC for identifying patients at risk of delirium. The random forest classifiers also exhibited strong performance, although slightly lower than that of the XGB model. Notably, both XGB and random forest classifiers maintained high predictive accuracy, indicating good model generalizability. In contrast, support vector classifier and logistic regression models showed substantially lower discriminative power. To further validate the clinical applicability of the XGB model, model performance was assessed using several evaluation metrics,including accuracy, sensitivity, positive predictive value, and negative predictive value (Table 4). Furthermore, the corresponding confusion matrices illustrating these metrics are displayed in Figure 3.

**Figure 4:**
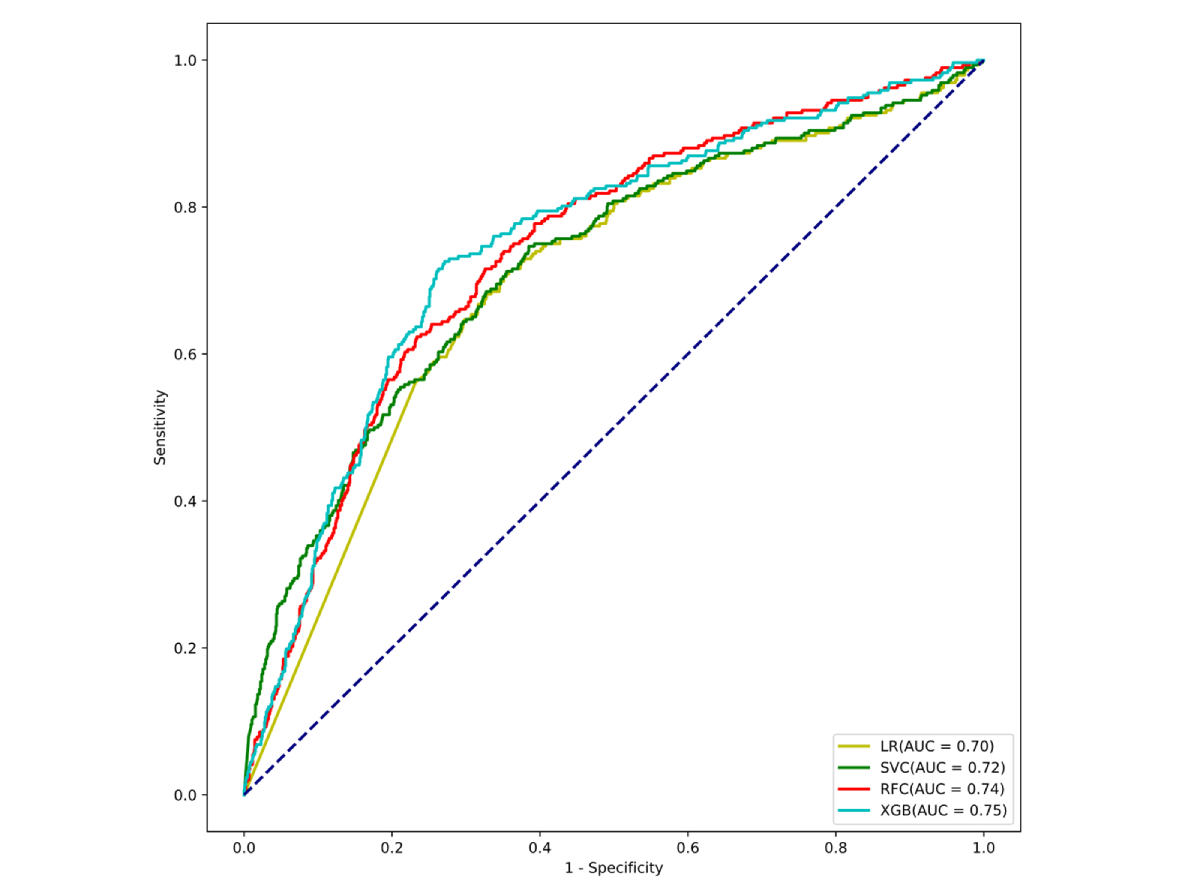
Receiver operating characteristic curves of various machine learning models evaluated on the external validation cohort. LR: logistic regression; RFC: random forest classifier; SVC: support vector classifier; XGB: extreme gradient boosting.

**Figure 5:**
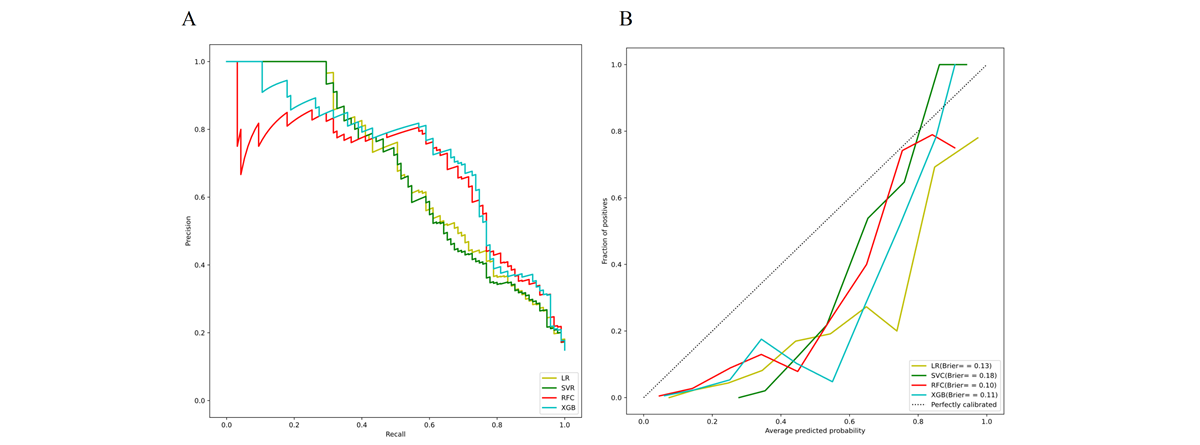
(A) Precision-recall curve. (B) Calibration curves and calculated Brier scores. LR: logistic regression; RFC: random forest classifier; SVC: support vector classifier;XGB: extreme gradient boosting.

The model was externally validated using the eICU-CRD, a large-scale critical care dataset incorporating records from 208 hospitals, to assess its performance on independent data. The XGB model maintained strong discriminative performance in ROC analysis, with high AUC values confirming its reliability across institutions (Figure 4). The model was further rigorously validated through precision-recall analysis and calibration curves (Figures 5A and B).

**Table 4:** Test set evaluation of machine learning model performance.

Model	Accuracy	Sensitivity	AUC^a^	PPV^b^	NPV^c^	*F*_1_-score
LR^d^	0.82	0.73	0.88	0.44	0.95	0.55
XGB^e^	0.83	0.77	0.91	0.47	0.95	0.58
SVC^f^	0.83	0.67	0.87	0.45	0.94	0.54
RFC^g^	0.88	0.75	0.90	0.58	0.95	0.65

^a^AUC: area under the receiver operating characteristic curve.

^b^PPV: positive predictive value.

^c^NPV: negative predictive value.

^d^LR: logistic regression.

^e^XGB: extreme boosting gradient.

^f^SVC: support vector classifier.

^g^RFC: random forest classifier.

### Variable Importance

**Figure 6:**
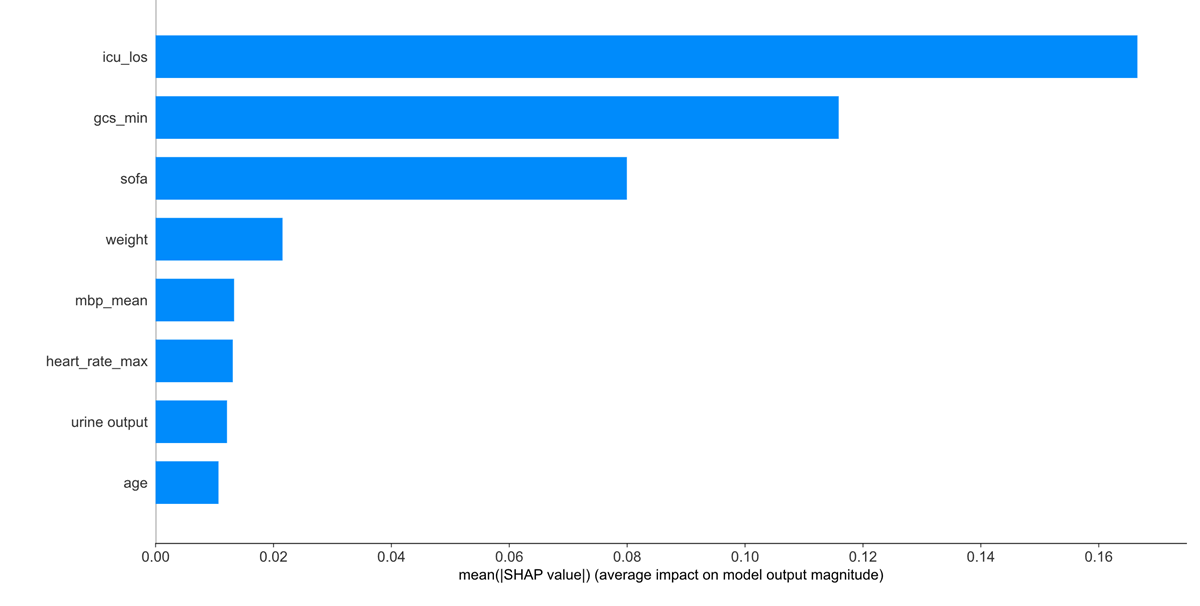
Variable contribution rankings estimated using the XGB model. GCS: Glasgow Coma Scale; ICU_los: the length time of ICU; MBP: mean blood pressure; SOFA: Sequential Organ Failure Assessment.

**Figure 7:**
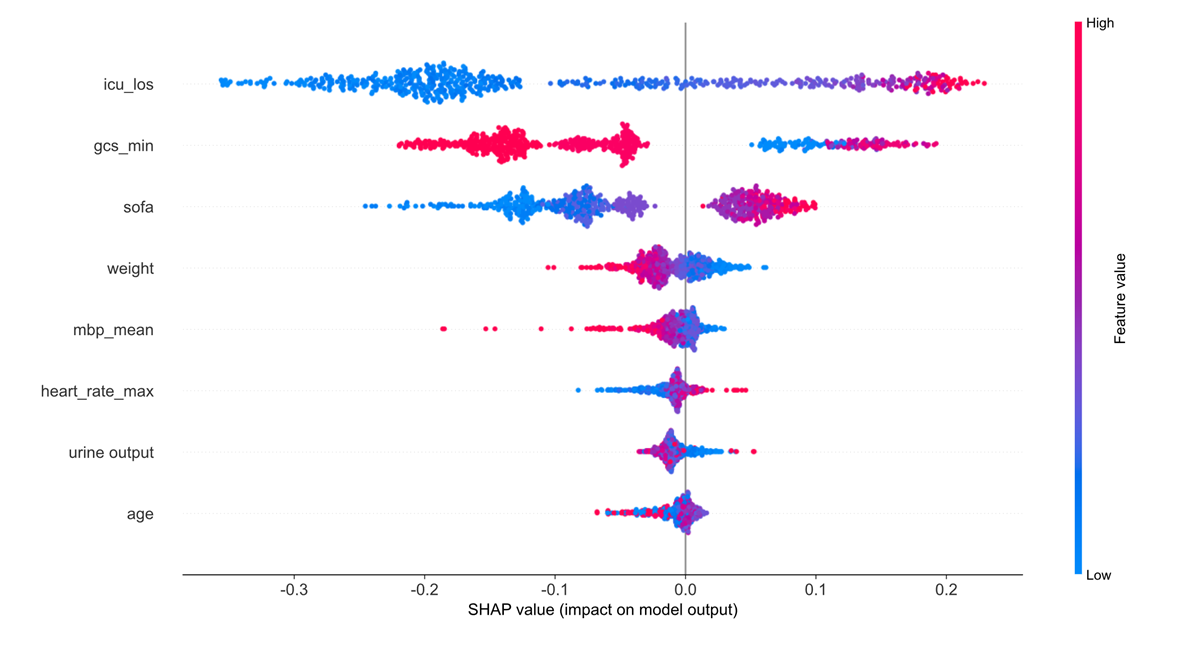
Shapley Additive Explanations (SHAP) summary plots illustrating feature contributions in the extreme gradient boosting (XGB) model. GCS: Glasgow Coma Scale; ICU_los: length of stay in the intensive care unit; MBP: mean blood pressure; SOFA: Sequential Organ Failure Assessment.

The predictive contribution of each variable to postoperative delirium varied among cardiac surgery ICU patients. The XGB algorithm was used to estimate variable importance and highlight the top predictors influencing model accuracy. The top predictors included length of stay in ICU, lowest GCS score, age, mean blood pressure, SOFA score, weight, heart rate and urine output (Figure 6). To further interpret the influence and directionality of these features on model predictions, we generated SHAP summary plots (Figure 7). These visualizations provide a detailed explanation of how individual features contributed to the model output, offering insights into their relative impact and potential clinical relevance.

## Discussion

### Principal Findings

In this large-scale retrospective study, we developed and validated an ML model to predict postoperative delirium, a common and burdensome complication after cardiac surgery, with reported incidence rates ranging from 18% to 52% [33-35]. The XGB-based model, trained on the MIMIC-IV 2.0 dataset, demonstrated excellent performance in internal validation (AUC=0.91) and maintained strong discriminative power in external validation using the eICU-CRD dataset (AUC=0.75). Key predictors identified by the model included length of stay in ICU, min GCS score, mean blood pressure, SOFA score, weight, urine output, heart rate, and age. As far as we are aware, this study presents one of the earliest ML models that are developed to predict delirium in a diverse population of cardiac surgery patients. Importantly, our model relies on routinely available clinical data from the first 24 hours of ICU admission, enabling early identification of high-risk patients throughout their ICU stay. This early-warning capability offers a critical window for timely intervention, allowing clinicians to implement targeted strategies at the earliest stages of delirium onset. The model’s high temporal relevance and wide applicability support its potential use in guiding personalized care plans and improving patient outcomes.

### Comparison With Previous Work

Among existing tools for predicting postoperative cognitive complications, the CAM-ICU score remains the most widely used [36-38]. However, its applicability may be limited in certain surgical contexts or patient subgroups. Some individual populations require more tailored analysis, and it is important to note that CAM-ICU may not capture the full spectrum of delirium severity, potentially compromising its construct validity [12,39,40]. Therefore, balancing feasibility and validity in specific clinical settings is essential. Our ML approach addresses these limitations by offering a data-driven, individualized risk assessment that adapts to patient heterogeneity and enables broader clinical applicability.

Furthermore, this study incorporated SHAP to enhance the interpretability of the XGB model. SHAP provides a transparent and traceable explanation framework for individualized risk prediction of delirium, allowing visualization of how each feature influences the model’s output in both direction and magnitude [41,42]. This helps clinicians better understand the rationale behind specific predictions. Through SHAP analysis, we identified a set of clinically relevant predictors closely associated with patient deterioration, including length of stay in ICU, lowest GCS score, age, mean blood pressure, SOFA score, weight, urine output, and heart rate.

The XGB model demonstrated superior predictive performance, its clinical value extends beyond accuracy—it lies in its ability to inform real-time decision-making. Future research should focus on integrating these key features into dynamic clinical monitoring and intervention workflows. For instance, early abnormalities in high-impact variables, such as lowest GCS score, mean blood pressure, or urine output could trigger automated alerts within electronic health record systems, enabling real-time risk stratification. Additionally, individualized SHAP-based explanations could support patient-centered clinical strategies, facilitating a closed-loop “early warning–targeted intervention” model. This approach may ultimately enhance early prevention and improve outcomes in postoperative delirium management.

### Strengths and Limitations

This study has several notable strengths. First, model development was based on large scale, real-world data from 2 publicly available critical care databases (MIMIC-IV 2.0 and eICU-CRD), which offer extensive clinical information and robust sample sizes, thereby increasing the trustworthiness and general applicability of our findings. Second, as far as we are aware, this is the earliest known study to apply ML techniques for predicting postoperative delirium in a broad cardiac surgery cohort. This tailored approach enables more accurate risk stratification by accounting for individual variability across surgical subtypes. One notable strength of our model lies in its exclusive use of routinely available clinical data obtained within the first day of ICU stay, eliminating the need for additional or specialized testing. The use of early time-series features to quantify dynamic changes in key physiological indicators allows real-time risk estimation with high clinical practicality and timeliness. From a translational perspective, the model demonstrated a sensitivity of 77% in identifying high-risk patients early, providing a valuable window for initiating preventive interventions. These proactive measures have the potential to reduce ICU length of stay, lower per-patient hospitalization costs, and minimize readmission risk, ultimately interrupting the cascade of adverse outcomes often associated with postoperative delirium. Finally, by focusing on the first day of ICU data, the model creates a strategic opportunity for timely clinical action, supporting the implementation of early, targeted strategies aimed at mitigating symptom progression and reducing complication rates.

Our study has several limitations. First, we acknowledge that our findings may be racially biased against White patients and the applicability to other populations may be limited due to the database being derived from Western countries. Second, the use of open public databases to obtain data may introduce missing data bias, which is unavoidable. Third, as this study is retrospective and observational in nature, selection bias is inevitable. Specifically, the collection of retrospective data depends on existing medical records that may vary in completeness and accuracy due to differences in clinical documentation practices and data entry habits. Such inconsistencies can result in missing or inaccurate key clinical information, which in turn affects the quality of data used for model training. Moreover, retrospective studies lack the ability to actively intervene in or control the research process, making it difficult to eliminate the influence of confounding variables. This limitation weakens the strength of causal inferences drawn from the analysis. Additionally, our model has not yet undergone prospective clinical validation, which limits its current applicability in real-world clinical practice. Prospective validation, ideally conducted under well-controlled trial conditions and standardized workflows, is essential for evaluating the predictive performance and robustness of the model in diverse clinical settings. Without this step, there remains a risk that the model’s actual performance may deviate from the retrospective findings, particularly when applied to different health care systems or patient populations.

### Conclusions

We constructed and assessed an effective predictive model targeting postoperative delirium in patients admitted to the ICU after cardiac surgery. This model leverages routinely available patient information from the initial 24-hour ICU stay to estimate the risk of delirium throughout the hospitalization period. Given its reliance on readily available early-stage variables, the model has the potential to serve as a practical and accessible risk stratification tool for health care professionals and patients when selecting optimal cardiac treatment strategies.

## Abbreviations

AUC: area under the receiver operating characteristic curve
CAM-ICU: Confusion Assessment Method for the Intensive Care Unit
eICU-CRD: eICU Collaborative Research Database
GCS: Glasgow Coma Scale
ICU: intensive care unit
MIMIC-IV 2.0: Medical Information Mart for Intensive Care IV version 2.0
ML: machine learning
ROC: receiver operating characteristic
SHAP: Shapley Additive Explanations
SOFA: Sequential Organ Failure Assessment
XGB: extreme gradient boosting

## Data Availability

The datasets used and analyzed during this study are available from the corresponding author on reasonable request.
